# Title-plus-abstract versus title-only first-level screening approach: a case study using a systematic review of dietary patterns and sarcopenia risk to compare screening performance

**DOI:** 10.1186/s13643-023-02374-3

**Published:** 2023-11-13

**Authors:** Lynn Teo, Mary E. Van Elswyk, Clara S. Lau, Christopher J. Shanahan

**Affiliations:** 1 Teo Research Consulting, Portland, ME USA; 2 Van Elswyk Consulting, Inc, Clark, CO USA; 3 National Cattlemen’s Beef Association, a contractor to the Beef Checkoff, 1275 Pennsylvania Avenue NW, Suite 801, Washington, D.C 20004 USA; 4 Unleash Creatives, LLC, Reynoldsburg, OH USA

**Keywords:** Systematic review, Contingency tables, Methodology, Dietary pattern, Citation screening

## Abstract

**Background:**

Conducting a systematic review is a time- and resource-intensive multi-step process. Enhancing efficiency without sacrificing accuracy and rigor during the screening phase of a systematic review is of interest among the scientific community.

**Methods:**

This case study compares the screening performance of a title-only (Ti/O) screening approach to the more conventional title-plus-abstract (Ti + Ab) screening approach. Both Ti/O and Ti + Ab screening approaches were performed simultaneously during first-level screening of a systematic review investigating the relationship between dietary patterns and risk factors and incidence of sarcopenia. The qualitative and quantitative performance of each screening approach was compared against the final results of studies included in the systematic review, published elsewhere, which used the standard Ti + Ab approach. A statistical analysis was conducted, and contingency tables were used to compare each screening approach in terms of false inclusions and false exclusions and subsequent sensitivity, specificity, accuracy, and positive predictive power.

**Results:**

Thirty-eight citations were included in the final analysis, published elsewhere. The current case study found that the Ti/O first-level screening approach correctly identified 22 citations and falsely excluded 16 citations, most often due to titles lacking a clear indicator of study design or outcomes relevant to the systematic review eligibility criteria. The Ti + Ab approach correctly identified 36 citations and falsely excluded 2 citations due to limited population and intervention descriptions in the abstract. Our analysis revealed that the performance of the Ti + Ab first-level screening was statistically different compared to the average performance of both approaches (Chi-squared: 5.21, *p* value 0.0225) while the Ti/O approach was not (chi-squared: 2.92, *p* value 0.0874). The predictive power of the first-level screening was 14.3% and 25.5% for the Ti/O and Ti + Ab approaches, respectively. In terms of sensitivity, 57.9% of studies were correctly identified at the first-level screening stage using the Ti/O approach versus 94.7% by the Ti + Ab approach.

**Conclusions:**

In the current case study comparing two screening approaches, the Ti + Ab screening approach captured more relevant studies compared to the Ti/O approach by including a higher number of accurately eligible citations. Ti/O screening may increase the likelihood of missing evidence leading to evidence selection bias.

**Systematic review registration:**

PROSPERO Protocol Number: CRD42020172655.

**Supplementary Information:**

The online version contains supplementary material available at 10.1186/s13643-023-02374-3.

## Background

Systematic reviews and meta-analyses are a necessary foundation of evidence-based public health recommendations as they help synthesize vast amounts of research to aid in evidence-based healthcare decisions and policy making [1, 2]. Systematic reviews are rigorous and time- and resource-intensive processes involving many steps and may often take years to complete [3, 4].

A key step in the systematic review process is during the screening phase when one needs to make a judgement on whether a study should be included or excluded based on the pre-determined eligibility criteria. While conventional practice is to screen both the title and abstract (i.e., title-plus-abstract (Ti + Ab)) at the initial screening phase [2, 5, 6], there has been interest in using a title-only (Ti/O) based screening approach to expedite the process [7–9]. The aim of this case study is to compare the relative screening performance of a title-only (Ti/O) screening approach to the more conventional title-plus-abstract (Ti + Ab) screening approach during first-level screening of citations using a systematic review protocol designed to investigate the relationship between dietary patterns and risk of sarcopenia using disease endpoints and risk factors [10].

## Methods

The main outcomes of the current study were designed to quantitatively compare the relative accuracy, sensitivity, specificity, and positive predictive power of the Ti/O versus Ti + Ab approaches; as well as to investigate qualitative reasons for incorrect exclusions of citations of each screening approach. To test the screening performance of the two screening approaches for eligibility for systematic review inclusion, one investigator followed the Ti/O screening approach, and two other investigators separately followed the Ti + Ab screening approach.

### Screening and study selection

The same pre-defined eligibility criteria, according to population, intervention, comparator, outcome, and study design (PICOS) were used for both Ti/O and Ti + Ab screening approaches and are reported in Additional file 1.

During the first-level screening, two investigators with systematic review expertise (L.T. and M.V.E.) independently followed the Ti + Ab approach and a third investigator with subject matter expertise (C.L.) independently followed the Ti/O approach. For training purposes, each screener followed their assigned screening approach on a subset of 1,998 citations (out of a total of 8,526 citations) until an inter-rater agreement reached over 90%. After this training period, C.L. screened all remaining citations using the Ti/O approach, and L.T. and M.V.E. divided the same remaining citations and screened using the Ti + Ab approach. Endnote was used to track the screening approach for each individual screener. At the conclusion of first-level screening, the full team used a Ti + Ab approach to re-screen all studies which passed the first-level screening, regardless of the screening approach, according to the eligibility criteria for the systematic review [10].

### Statistical analysis

To test the relative performance of the two first-level study screening approaches, contingency tables were developed for each screening approach (Table 1) and compared against each other using the R statistical analysis package and Microsoft Excel [11]. A contingency table is a simple 2 × 2 matrix that maps the performance of a given screening approach where one of the axes indicates a study’s eligibility qualification status (e.g., whether a study should have been included/excluded) and the other axis indicates the study’s actual eligibility status (e.g., whether a study has actually been included/excluded) as determined by the reviewer [12].

**Table 1:** Contingency table and associated variables used to assess each study screening approach^a^

	Qualified studies	Unqualified studies	Potential studies
Included studies	Included qualified studies or Included ‘Trues’ T	Included unqualified studies or Included ‘Falses’ F	Potential included studies T + F
Excluded studies	Excluded qualified studies or Excluded ‘Trues’ Q-T	Excluded unqualified studies or Excluded ‘Falses’ E–F	Potential excluded studies Q + E-(T + F)
Total studies	Total qualified studies or Total ‘Trues’ Q	Total unqualified studies or Total ‘Falses’ E	Total potential studies Z = Q + E

^a^A contingency table is a simple 2 × 2 matrix where one of the axes indicates a study’s eligibility qualification status (e.g., whether a study should have been included/excluded) and the other axis indicates the study’s actual eligibility status (e.g., whether a study has actually been included/excluded) as determined by the reviewer. From this matrix, the following key metrics can be calculated to help determine screening performance [13]:

*• Sensitivity* = *T / Q* (This measure describes how effective the screening approach is at correctly identifying which studies to *include* and how ineffective it is at *excluding qualified studies*)

*• Specificity* = *(E—F) / E* (This measure describes how effective the screening approach is at correctly identifying which studies to *exclude* and how ineffective it is at *including unqualified studies*)

*• Accuracy* = *((T / Q)* + *((E—F) / E)) / Z* (This measure describes how effective the screening approach is at correctly identifying which studies to *include and exclude overall*)

*• Predictive Power* = *T / (T* + *F)* (This measure describes how effective overall the screening approach is at including qualified studies)

Eligible studies (i.e., trues) that either passed or did not pass the first-level screening were defined as “included trues” and “excluded trues”, respectively. Eligible studies were studies ultimately included in the final analysis of the systematic review [10]. Ineligible studies (i.e., falses) that either passed or did not pass the first-level screening were defined as “included falses” and “excluded falses”, respectively. Ineligible studies were studies ultimately not included in the final analysis of the systematic review [10]. Table 1 provides a graphical representation of a contingency table used to structure and assess each screening approach’s predictive power.

Contingency tables were used to determine which screening approach is relatively more effective at maximizing the number of correct judgments and/or minimizing the number of incorrect judgements. The relative accuracy of a given screening approach (i.e., ability to correctly identify and classify both qualified or unqualified studies) is a function of the relative sensitivity of the approach or maximizing the number of correct judgments (e.g., “included trues” and “excluded falses”) and the relative specificity of the approach or minimizing the number of incorrect judgments (e.g., “excluded trues” and “included falses”). The sensitivity of the screening approach is the percentage of the citations ultimately included in the systematic review that were correctly identified at the first-level screening stage using the specific screening approach. The specificity reflects the screening approach’s ability to minimize incorrect judgments allowing for the correct identification and exclusion of unqualified citations. The predictive power is the percentage of included studies correctly predicted to be eligible.

Mathematically, the accuracy of a specific screening approach $i$ was deduced using the following equation:

1 $${A}_{i}=\frac{\left(\frac{{T}_{i}}{{Q}_{i}} + \frac{{{E}_{i}-F}_{i}}{{E}_{i}}\right)}{Z}$$

where ${A}_{i}$ is the accuracy fraction of screening approach $i$, $Z$ is the total potential number of citations included at the initial screening level by one or more reviewers, ${T}_{i}$ is the total number of “trues” included by the reviewer given the study screening approach, ${Q}_{i}$ is the total number of “trues” in the study set $Z$, ${F}_{i}$ is the total number of “falses” included by the reviewer given the study screening approach, and ${E}_{i}$ is the total number of “falses” in the study set $Z$. The fraction $\frac{{T}_{i}}{{Q}_{i}}$ is the sensitivity of the screening approach and the fraction $\frac{{{E}_{i}-F}_{i}}{{E}_{i}}$ is the screening approach’s specificity fraction. In addition, the predictive power ${P}_{i}$ ratio of each screening approach was determined from the contingency table and is calculated as follows:

2 $${P}_{i}={T}_{i}/\left({T}_{i}+{F}_{i}\right).$$

${P}_{i}$ describes how well the first-level screening approach was at correctly predicting the qualified studies included or “included trues.”

To test whether a given screening approach was statistically more effective in terms of sensitivity and specificity, and thus relative accuracy and predictive power, each screening approach was compared to a composite or average contingency table of both screening approaches to calculate the differences in sensitivity and specificity and then Chi-squared test statistics and associated p-values for each comparison were calculated. If a Chi-squared test of a given screening approach’s sensitivity and specificity measurements are found to be statistically significant and higher in value compared to the other screening approach, then this implies that the given screening approach is likely more effective at obtaining an accurate outcome..

## Results

### Quantitative results

**Fig. 1:**
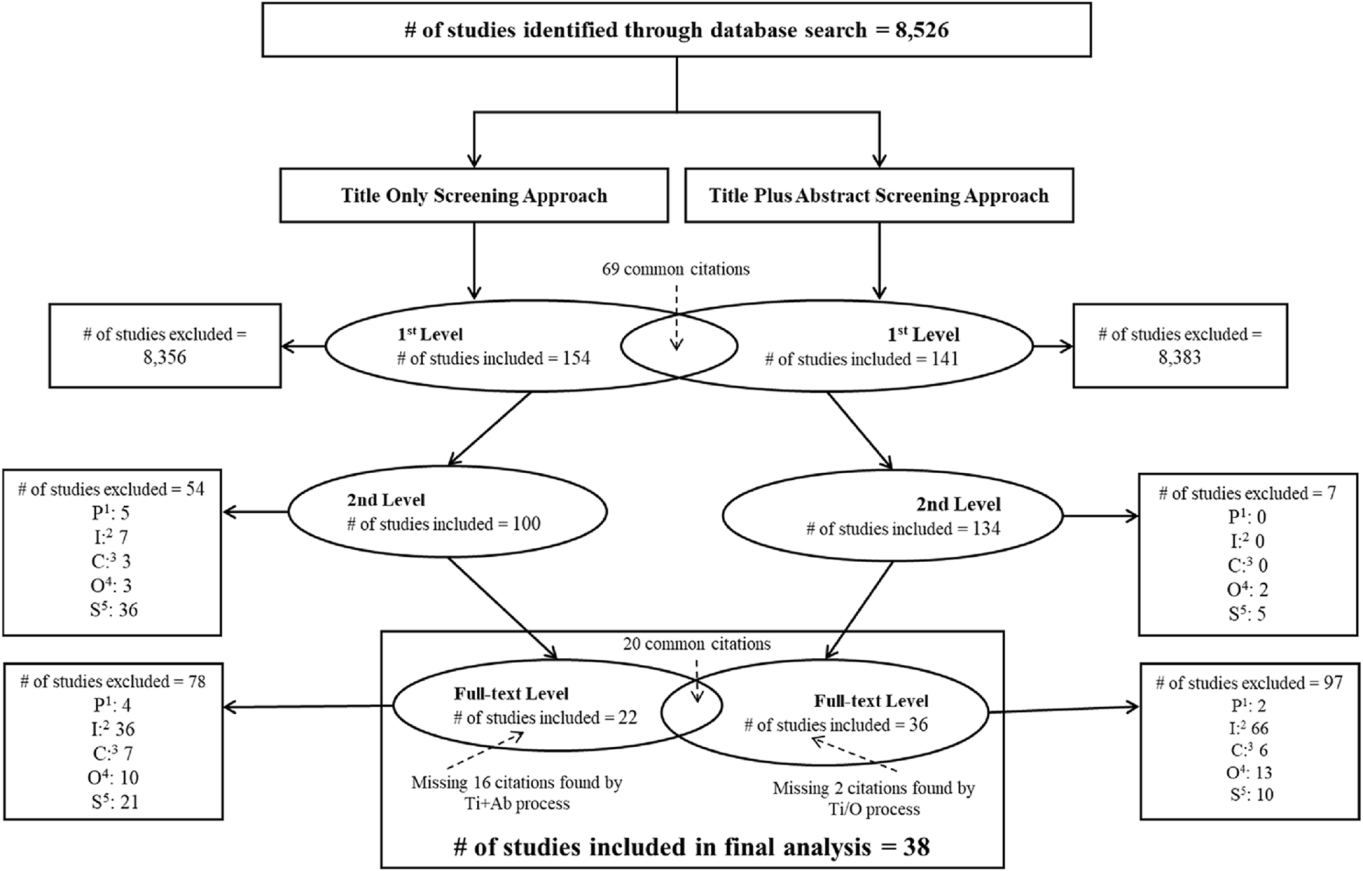
Systematic review flow chart comparing screening results between a title-only (Ti/O) to a title-plus-abstract (Ti + Ab) screening approach at the first level. 1st level: first-level screening where two investigators independently followed a Ti + Ab screening approach, and a 3rd investigator followed a Ti/O screening approach. 2nd level: second-level screening where the full team rescreened all studies which passed the first-level screen using a Ti + Ab screening approach. Full-text level: full-text screening where the full team screened all studies which passed the second-level screen ^1^Population; ^2^Intervention; ^3^Comparison; ^4^Outcome; ^5^Study design

When comparing the results of the first-level screening to the final analysis, the Ti/O screening approach resulted in missing 16 of the 38 citations that were eventually included in the final systematic review (Fig. 1), resulting in an excluded “trues” rate of 42.1% (Table 2). The Ti/O screening approach’s sensitivity was 57.9%; with 22 of the 38 studies ultimately included in the systematic review correctly identified using the Ti/O approach. The specificity of the Ti/O approach was 98.4%, allowing the correct identification and exclusion of 8356 unqualified citations out of a total of 8526 citations, and an accuracy score of 98.3%. With a predictive power of 14.3%, the Ti/O screening approach correctly predicted only 14.3% of the included studies versus the average predictive power of 19.7%. The overall screening performance of the Ti/O approach was not statistically significantly different from the average performance as reported in Table 2 (chi-squared statistic = 2.92; *p* value = 0.874; Table 2C).

**Table 2:** Study screening approach contingency tables and performance results

A. Title-only contingency table and performance results			
	*Qualified*	*Unqualified*	*Potential*
*Included*	22	132	154
*Excluded*	16	8356	8372
*Total*	38	8488	8526
*Sensitivity (included ‘trues’)*			57.89%
*Excluded ‘trues’ rate*			42.11%
*Specificity*			98.44%
*Accuracy*			98.26%
*Predictive power*			14.3%
*Chi-squared statistic*^a^			2.9208
*p value*^a^			0.874
B. Title + abstract contingency table and performance results			
	*Qualified*	*Unqualified*	*Potential*
*Included*	36	105	141
*Excluded*	2	8383	8,385
*Total*	38	8488	8,526
*Sensitivity (included ‘trues’)*			94.74%
*Excluded ‘trues’ rate*			5.26%
*Specificity*			98.76%
*Accuracy*			98.75%
*Predictive power*			25.5%
*Chi-squared statistic*^a^			5.208
*p value*^a^			0.0225^c^
C. Average performance results contingency table^b^			
	*Qualified*	*Unqualified*	*Potential*
*Included*	29	119	148
*Excluded*	9	8370	8,379
*Total*	38	8488	8,526
*Average sensitivity (included ‘trues’)*			76.32%
*Excluded ‘trues’ rate*			23.68%
*Average specificity*			98.60%
*Average accuracy*			98.50%
*Average predictive power*			19.7%

^a^Chi-squared statistics and associated *p* values were calculated to compare each first-level screening approach against the average performance of either screening approach

^b^The average performance is defined as the average screening performance across both screening approaches taken together. For example, it is expected that 29 studies (or (22 + 36)/2) = 29) will be correctly identified and included in the next stage of evaluation

^c^Indicates that the difference in screening performance versus the average performance results is statistically different with + 95% confidence

The performance of the Ti + Ab first-level screening approach resulted in missing 2 of the 38 citations that were eventually included in the final systematic review (Fig. 1), resulting in an “excluded trues” rate of 5.3% (Table 2). With 36 of the 38 qualified studies correctly identified using the Ti + Ab approach at the first-level screening stage, the sensitivity of 94.7% was higher than that of the Ti/O approach (Table 2 and Fig. 1). The specificity of the Ti + Ab approach was 98.8% and an accuracy score of 98.75%. At 25.5%, the predictive power of the Ti + Ab approach was higher than the average screening performance of 19.7%. Overall, the performance of the Ti + Ab first-level screening approach is significantly different from the average performance (chi-squared statistic = 5.21; *p* value = 0.0225; Table 2C and Fig. 1).

### Qualitative results

The Ti/O approach mistakenly excluded 16 qualified (“excluded trues”) citations that were included by the Ti + Ab approach and ultimately included in the final systematic review. A closer retrospective look at the “excluded trues” by the Ti/O approach revealed that 14 titles were lacking a clear study design element. Further, 13 study titles made no mention of an outcome measure directly related to the systematic review’s eligibility criteria (see Additional file 2).

Of the 141 citations passed by the Ti + Ab approach at first-level screening, 36 citations were ultimately included for the systematic review. Two citations were falsely excluded where the description of the population was not clear in one abstract [14] and the description of protein quartiles was not clearly described as being outside the acceptable macronutrient distribution range (AMDR) in the other abstract [15].

## Discussion

This case study found that Ti/O screening performed in accordance with statistical expectations while Ti + Ab performed significantly better than was expected. It should be noted that the Chi-squared statistic only tests whether the screening approach’s performance is statistically different from a composite or average level of performance. It does not indicate how or why there is a difference in performance. Only a comparison of the summary performance statistics from the test and average contingency tables (i.e., specificity, sensitivity, accuracy, and predictive power) can address the questions of how and why there is a relative difference in performance. This case study found the Ti + Ab screening approach at the first-level screening phase had higher predictive power due to being more effective at finding qualified studies to include in the next stage of evaluation compared to the Ti/O screening approach. This suggests that reviewing both the title and abstract during first-level review stage resulted in a more effective screening performance.

Our case study demonstrated that both the Ti/O and Ti + Ab first-level screening approaches achieved high specificity (98.4% and 98.8%, respectively), due to the small base of qualified studies within the total number of citations included at the initial screening level. In our case, high specificity was easy to achieve as the actual prevalence of eligible studies in the initial search results is relatively small (36/8526 = 0.4%) allowing for a high rate of correct identification and exclusion of unqualified studies by both approaches. Likewise, both the Ti/O and Ti + Ab first-level approaches also achieved high accuracy (98.3% and 98.8%).

The Ti + Ab approach’s predictive power of 25.5% was observed to be higher than the Ti/O approach’s power of 14.3%. This suggests that more information assessed at the first-level screening will likely increase the number of studies correctly predicted to be eligible; thus less time will be required for the next stage of evaluation. This also suggests that despite the Ti/O approach including comparably more studies for the next stage evaluation compared to the Ti + Ab approach (154 citations versus 141 citations, respectively, Table 2), the predictive power of the Ti/O screening approach is still lower and thus less reliable for identifying qualified studies. Further, compared to the Ti/O approach, the Ti + Ab approach had a much lower excluded “trues” rate (5.3% vs. 41.1%) and higher sensitivity (57.9% vs. 94.7%) highlighting that the latter approach is more likely to capture studies of interest and less likely to pass unqualified studies to the full-text level, potentially saving both time and financial resources. Methodologies that systematically lead to missing relevant articles result in evidence selection bias, a bias that occurs when all available data on a topic has not been identified [16]. This can impact the synthesis of evidence and bias the resulting conclusions, in a direction inconsistent with the true association [2].

Our findings parallel a previous study which compared the differences between Ti/O and Ti + Ab screening approaches where Mateen et al. [8], found that Ti + Ab screening achieved higher precision compared to the Ti/O approach and required the review of fewer full-text articles compared to the Ti/O screening. However, the authors also suggest that Ti/O screening was possibly more efficient than Ti + Ab screening because of the expected time saved from not reading the abstracts of unqualified studies, even though more time was required during the full-text review stage. The authors do admit that they did not measure relative screening times and thus could not provide substantiation of their time-savings hypothesis.

Choosing a screening approach may be based on the nature of the eligibility criteria. Titles alone may not have enough information to make predefined PICOS eligibility judgments. More detailed eligibility criteria may increase the likelihood that titles alone will not be enough to reveal if a study qualifies. Intermediate markers of sarcopenia risk (i.e., skeletal muscle mass, muscle strength, muscle performance) may not have been explicitly mentioned in titles but only relayed in the abstract or the full text. For example, in this case study, four titles that mentioned “frailty” were screened out using Ti/O because this was not an outcome of interest but the Ti + Ab screening provided the opportunity to review more detailed methodology and information regarding measurement of secondary outcomes that were consistent with the eligibility criteria. Information in the abstracts revealed that frailty can be determined by a variety of outcomes including gait speed and hand grip which were also eligible measurements of muscle strength and muscle performance in our protocol (see Additional file 2). Zhu et al.’s “A Prospective Investigation of Dietary Intake and Functional Impairments Among the Elderly [17]” does not mention investigating impairment in walking capability, a measure of muscle performance, in its title but this information is available in the abstract. To capture these outcomes of interest, Ti + Ab screening would become a necessary next step of Ti/O screening, and erring on the side of inclusion to screen more full texts may be needed for eligibility determination. There are situations when the title or sometimes even the abstract of a paper does not contain all the PICOS information necessary to predict whether the citation should be included. This is particularly true for studies published previous to reporting guidelines: the CONSORT Statement [18], the reporting guidelines for randomized controlled trials was developed in 1996; and QUORUM, the predecessor to the PRISMA Statement, the reporting guidelines for systematic reviews and meta-analyses was developed in 1999 [19]. In the current review, the information needed to assess whether a dietary pattern based on a macronutrient distribution, where at least one macronutrient had to be outside of AMDR, would often not be present in the title alone (e.g., “Adult macronutrient intake and physical capability in the MRC National Survey of Health and Development [20]”) nor sufficiently specified in the abstract. In this case, erring on the side of inclusion for full-text review during screening would be a viable strategy to avoid missing relevant citations.

The Ti + Ab approach to first-level screening in this study mistakenly excluded two citations, one per Ti + Ab reviewer, that were ultimately included in the final analysis, despite having access to the abstract. Due to the complexity of the eligibility criteria in this case study, it can be debated that each citation should have been reviewed in tandem per screening approach to avoid excluded trues. It is common for teams to debate whether some studies fit the eligibility criteria, even at the full-text level, as in the case of these two studies (see Additional file 2).

Should Ti/O prove effective and time-saving as a screening approach, reporting guidelines should consider recommending study titles to include all PICOS elements, as currently the abstract is used for this information. Implementing revised reporting guidelines would further require reconsideration by journals of title character limitations which currently prove a challenge when attempting to include all PICOS elements. Currently, both the current CONSORT Statement [21] for randomized controlled trials, and the PRISMA Statement [22] for systematic reviews and meta-analyses recommend that the study design is explicit in the title. CONSORT explicitly requires PICO to be included in the journal and conference abstracts [23] and the PRISMA Statement recommends that the study design is explicit in the title and that the eligibility criteria (which usually reflects PICO) are reported in the abstract [22]. Additionally, the Journal of the American Medical Association as well as the Annals of Internal Medicine requires the type of study design (i.e., clinical trials, meta-analyses, and systematic reviews) as part of the publication’s title [24, 25]. While not an exhaustive review of the topic, these examples suggest that standard reporting guidelines and journal requirements for titles may not readily support the use of the Ti/O screening approach.

This case study has limitations. First, it would have been more methodologically optimal to have two investigators conducting Ti/O first-level screening in tandem for consistency with the Ti + Ab first-level screening. Further, there were differences in backgrounds in investigating teams for the two approaches: the Ti/O approach was performed by an investigator with subject matter expertise while Ti + Ab approach was performed by two investigators with systematic review expertise. Second, the average time to conduct the first-level screening using either approach was not measured. This would have been useful information as time taken is essential to reflect both efficiency as well as cost. Time was not tracked as this case study was a secondary focus to the original systematic review. This being said, we would recommend tracking time in future similar investigations. Lastly, this case study is based on the manual screening of citations and may not be generalizable to screening approaches using artificial intelligence (A.I.) or automated screening technology (e.g., DistillerSR or Covidence) to lessen the workload attributed to screening. On the other hand, this case study has strengths. First, this case study utilizes eligibility criteria developed by (although endorsement by not implied) the United States Department of Agriculture’s Nutrition Evidence Systematic Review team (NESR) [10, 26, 27], government experts that specialize in conducting food- and nutrition-related systematic reviews. Most recently, NESR implemented a Ti/O approach at first-level screening for 33 original systematic reviews conducted to support the 2020 Dietary Guidelines Advisory Committee, which informed the development of the 2020–2025 Dietary Guidelines for Americans [28, 29]. Second, the results of this case study are supported by two systematic reviews that have both been recently peer-reviewed and published [10, 26]. Lastly, this case study adds to the limited knowledge base of evaluating the difference in Ti/O compared to Ti + Ab screening approaches when conducting systematic reviews.

## Conclusions

In summary, this case study demonstrated that using the conventional Ti + Ab screening approach had better screening performance than the Ti/O approach. If the final systematic review had relied only on Ti/O screening approach, 16 citations may have been erroneously excluded. Conducting an effective systematic review requires researchers to balance both researcher efficiency with a screening approach that maximizes eligible study inclusion to help reduce the risk of evidence selection bias and ensure a comprehensive evidence base. Avoidance of evidence selection bias stemming from missing relevant evidence is important to consider when conclusions of a systematic review are often used by researchers, clinicians, and key stakeholders to inform the development of clinical guidelines and public health recommendations.

### Supplementary Information

**Additional file 1. **Description of population, intervention, comparator, outcome, and study design (PICOS) criteria for the research question, “What is the relationship between dietary patterns and risk of sarcopenia?”

**Additional file 2. **Studies included in final systematic review (Van Elswyk et al., 2022) [10] as determined by each screening approach, passed at first-level screening.

## Data Availability

Data described in the manuscript will be made available upon request from Dr. Van Elswyk (e-mail: mveconsulting@q.com).

## Abbreviations

AMDR: Acceptable Macronutrient Distribution Range
NESR: United States Department of Agriculture’s Nutrition Evidence Systematic Review team
PICOS: Population, intervention, comparator, outcome, and study design
RCT: Randomized controlled trial
Ti + Ab: Title-plus-abstract
Ti/O: Title-only
