# Supplementary material for: Title-plus-abstract versus title-only first-level screening approach: a case study using a systematic review of dietary patterns and sarcopenia risk to compare screening performance
Source: Syst Rev. 2023 Nov 13;12:211. doi: 10.1186/s13643-023-02374-3 (PMC10644647; doi:10.1186/s13643-023-02374-3)
Supplement: Supplementary file 1 — Additional file 1. Description of population, intervention, comparator, outcome, and study design (PICOS) criteria for the research question, “What is the relationship between dietary patterns and risk of sarcopenia?” [file 13643_2023_2374_MOESM1_ESM.docx]

**Additional file 1.** Description of population, intervention, comparator, outcome, and study design (PICOS) criteria for the research question, “What is the relationship between dietary patterns and risk of sarcopenia?”

| **Parameter** | **Eligibility criteria** |
| --- | --- |
| **P**opulation | Human subjects ≥19 years at time of outcome who were healthy and/or at risk for chronic disease, not pregnant or lactating, living in countries ranked as high or higher human development. (Human development classification based on human development index rank from the year the study intervention occurred, or data were collected. Available from <http://hdr.undp.org/en/data>. Rank higher than 110 indicative of medium or lower human development index).  Study populations composed of a mixed population of healthy, at risk, and diseased subjects, but not exclusively diagnosed with a disease or with low skeletal muscle mass, low muscle strength, low muscle performance, or sarcopenia were included |
| **I**ntervention | Consumption of and/or adherence to a dietary pattern (measured as an index/score; factor or cluster analysis; reduced rank regression; having a macronutrient with at least one macronutrient outside the acceptable macronutrient distribution range (AMDR); or description of dietary pattern, including at a minimum, the foods and beverages included in pattern)  Outside AMDR: <45% or >65% of energy from carbohydrate; < 20% or >35% of energy from fat; or <10% or >35% of energy from protein.  Interventions for weight loss or examining dietary supplements or single foods as a macronutrient source (i.e., nuts) were not included. |
| **C**omparison | Consumption of and/or adherence to a different dietary pattern, varying levels of adherence to a dietary pattern, or different macronutrient proportions |
| **O**utcome | Intermediate markers of sarcopenia risk (i.e., measures of skeletal muscle mass, muscle strength, muscle performance) and/or incidence of sarcopenia or severe sarcopenia |
| **S**tudy design | Peer-reviewed controlled trials or observational trials (prospective cohort studies, retrospective cohort studies, nested case-control studies and case-control) published in English |
