# Supplementary material for: Title-plus-abstract versus title-only first-level screening approach: a case study using a systematic review of dietary patterns and sarcopenia risk to compare screening performance
Source: Syst Rev. 2023 Nov 13;12:211. doi: 10.1186/s13643-023-02374-3 (PMC10644647; doi:10.1186/s13643-023-02374-3)
Supplement: Supplementary file 2 — Additional file 2. Studies included in final systematic review (Van Elswyk et al., 2022) [10] as determined by each screening approach, passed at first-level screening. [file 13643_2023_2374_MOESM2_ESM.docx]

**Additional file** **2.** Studies included in final systematic review (Van Elswyk et al., 2022^1^) as determined by each screening approach, passed at first-level screening

| **Study Citation** | **Title-Only** | **Title+Abstract** |
| --- | --- | --- |
| Agarwal P, Wang Y, Buchman AS, Bennett DA, Morris MC. Dietary Patterns and Self-reported Incident Disability in Elderly. J Gerontol A Biol Sci Med Sci. 2018;74(8):1331-7. |  | X |
| Baker ME, DeCesare KN, Johnson A, Kress KS, Inman CL, Weiss EP. Short-Term Mediterranean Diet Improves Endurance Exercise Performance: a Randomized-Sequence Crossover Trial. J Am Coll Nutr. 2019;38(7):597-605. | X | X |
| Birnie K, Ben-Shlomo Y, Gunnell D, Ebrahim S, Bayer A, Gallacher J, Holly JM, Martin RM. Childhood milk consumption is associated with better physical performance in old age. Age Ageing. 2012;41(6):776-84. |  | X |
| Bishop NJ, Zuniga KE, Lucht AL. Latent Profiles of Macronutrient Density and their Association with Mobility Limitations in an Observational Longitudinal Study of Older U.S. Adults. J Nutr Health Aging. 2018;22(6):645-54. | X | X |
| Castaneda C, Charnley JM, Evans WJ, Crim MC. Elderly women accommodate to a low-protein diet with losses of body cell mass, muscle function, and immune response. Am J Clin Nutr. 1995;62(1):30‐9. |  | X |
| Cervo MM, Shivappa N, Hebert JR, Oddy WH, Winzenberg T, Balogun S, Wu F, Ebeling P, Aitken D, Jones G, et al. Longitudinal associations between dietary inflammatory index and musculoskeletal health in community-dwelling older adults. Clin Nutr. 2019 Feb 21 (Epub ahead of print; doi: 10.1016/j.clnu.2019.02.031). | X | X |
| Chan R, Leung J, Woo J. A Prospective Cohort Study to Examine the Association Between Dietary Patterns and Sarcopenia in Chinese Community-Dwelling Older People in Hong Kong. J Am Med Dir Assoc. 2016;17(4):336-42. | X | X |
| Dipla K, Makri M, Zafeiridis A, Soulas D, Tsalouhidou S, Mougios V, Kellis S. An isoenergetic high-protein, moderate-fat diet does not compromise strength and fatigue during resistance exercise in women. Br J Nutr. 2008;100(2):283‐6. | X | X |
| Germain L, Latarche C, Kesse-Guyot E, Galan P, Hercberg S, Briançon S. Does Compliance with Nutrition Guidelines Lead to Healthy Aging? A Quality-of-Life Approach. J Acad Nutr Diet. 2013;113(2):228-40. |  | X |

| **Study Citation** | **Title-Only** | **Title+Abstract** |
| --- | --- | --- |
| Gopinath B, Russell J, Flood VM, Burlutsky G, Mitchell P. Adherence to dietary guidelines positively affects quality of life and functional status of older adults. J Acad Nutr Diet. 2014;114(2):220-9. | X | X |
| Granic A, Jagger C, Davies K, Adamson A, Kirkwood T, Hill TR, Siervo M, Mathers JC, Sayer AA. Effect of dietary patterns on muscle strength and physical performance in the very old: Findings from the Newcastle 85+ study. PLoS One. 2016;11(3). | X | X |
| Granic A, Mendonça N, Sayer AA, Hill TR, Davies K, Siervo M, Mathers JC, Jagger C. Effects of dietary patterns and low protein intake on sarcopenia risk in the very old: The Newcastle 85+ study. Clin Nutr. 2020;39(1):166-73. | X | X |
| Hagan KA, Chiuve SE, Stampfer MJ, Katz JN, Grodstein F. Greater adherence to the alternative healthy eating index is associated with lower incidence of physical function impairment in the nurses' health study. J Nutr. 2016;146(7):1341-7. | X | X |
| Hagan KA, Grodstein F. The Alternative Healthy Eating Index and Physical Function Impairment in Men. J Nutr Health Aging. 2019;23(5):459-65. | X | X |
| Isanejad M, Sirola J, Mursu J, Rikkonen T, Kröger H, Tuppurainen M, Erkkilä AT. Association of the Baltic Sea and Mediterranean diets with indices of sarcopenia in elderly women, OSPTRE-FPS study. Eur J Nutr. 2018;57(4):1435-48. | X | X |
| Karlsson M, Becker W, Michaëlsson K, Cederholm T, Sjögren P. Associations between dietary patterns at age 71 and the prevalence of sarcopenia 16 years later. Clin Nutr. 2019;39(4):1077-84. | X | X |
| Laclaustra M, Rodriguez-Artalejo F, Guallar-Castillon P, Banegas JR, Graciani A, Garcia-Esquinas E, Lopez-Garcia E. The inflammatory potential of diet is related to incident frailty and slow walking in older adults. Clin Nutr. 2019 Jan 24 (Epub ahead of print; doi:10.1016/j.clnu.2019.01.013) |  | X |
| León-Muñoz LM, Guallar-Castillón P, López-García E, Rodríguez-Artalejo F. Mediterranean Diet and Risk of Frailty in Community-Dwelling Older Adults. J Am Med Dir Assoc. 2014;15(12):899-903. |  | X |
| León-Muñoz LM, García-Esquinas E, López-García E, Banegas JR, Rodríguez-Artalejo F. Major dietary patterns and risk of frailty in older adults: a prospective cohort study. BMC Med. 2015;13:11. |  | X |
| Mangano KM, Sahni S, Kiel DP, Tucker KL, Dufour AB, Hannan MT. Dietary protein is associated with musculoskeletal health independently of dietary pattern: The Framingham Third Generation Study. Am J Clin Nutr. 2017;105(3):714-22. | X |  |
| Meng X, Zhu K, Devine A, Kerr DA, Binns CW, Prince RL. A 5-year cohort study of the effects of high protein intake on lean mass and BMC in elderly postmenopausal women. J Bone Miner Res. 2009;24(11):1827-34. | X | X |
| Milaneschi Y, Bandinelli S, Corsi AM, Lauretani F, Paolisso G, Dominguez LJ, Semba RD, Tanaka T, Abbatecola AM, Talegawkar SA, et al. Mediterranean diet and mobility decline in older persons. Exp Gerontol. 2011;46(4):303-8. |  | X |
| Mulla UZ, Cooper R, Mishra GD, Kuh D, Stephen AM. Adult macronutrient intake and physical capability in the MRC National Survey of Health and Development. Age Ageing. 2013;42(1):81-7. |  | X |
| Parsons TJ, Papachristou E, Atkins JL, Papacosta O, Ash S, Lennon LT, Whincup PH, Ramsay SE, Wannamethee SG. Healthier diet quality and dietary patterns are associated with lower risk of mobility limitation in older men. Eur J Nutr. 2019;58(6):2335-43. | X | X |
| Perälä MM, Von Bonsdorff MB, Männistö S, Salonen MK, Simonen M, Kanerva N, Rantanen T, Pohjolainen P, Eriksson JG. The healthy Nordic diet predicts muscle strength 10 years later in old women, but not old men. Age Ageing. 2017;46(4):588-94. | X | X |
| Perälä MM, Von Bonsdorff M, Männistö S, Salonen MK, Simonen M, Kanerva N, Pohjolainen P, Kajantie E, Rantanen T, Eriksson JG. A healthy Nordic diet and physical performance in old age: Findings from the longitudinal Helsinki Birth Cohort Study. Br J Nutr. 2016;115(5):878-86. | X | X |
| Pérez-Tasigchana RF, León-Muñoz LM, López-García E, Banegas JR, Rodríguez-Artalejo F, Guallar-Castillón P. Mediterranean Diet and Health-Related Quality of Life in Two Cohorts of Community-Dwelling Older Adults. PLoS One. 2016;11(3):e0151596. |  | X |
| Pilis K, Pilis A, Stec K, Pilis W, Langfort J, Letkiewicz S, Michalski C, Czuba M, Zych M, Chalimoniuk M. Three-year chronic consumption of low-carbohydrate diet impairs exercise performance and has a small unfavorable effect on lipid profile in middle-aged men. Nutrients. 2018;10(12). | X |  |
| Pilleron S, Pérès K, Jutand MA, Helmer C, Dartigues JF, Samieri C, Féart C. Dietary patterns and risk of self-reported activity limitation in older adults from the Three-City Bordeaux Study. Br J Nutr. 2018;120(5):549-56. |  | X |
| Rahi B, Ajana S, Tabue-Teguo M, Dartigues JF, Peres K, Feart C. High adherence to a Mediterranean diet and lower risk of frailty among French older adults community-dwellers: Results from the Three-City-Bordeaux Study. Clin Nutr. 2018;37(4):1293-8. |  | X |
| Robinson SM, Westbury LD, Cooper R, Kuh D, Ward K, Syddall HE, Sayer AA, Cooper C. Adult Lifetime Diet Quality and Physical Performance in Older Age: Findings From a British Birth Cohort. J Gerontol A Biol Sci Med Sci. 2018;73(11):1532-7. | X | X |
| Shahar DR, Houston DK, Hue TF, Lee JS, Sahyoun NR, Tylavsky FA, Geva D, Vardi H, Harris TB. Adherence to mediterranean diet and decline in walking speed over 8 years in community-dwelling older adults. J Am Geriatr. Soc 2012;60(10):1881-8.. |  | X |
| Stefler D, Hu Y, Malyutina S, Pajak A, Kubinova R, Peasey A, Pikhart H, Rodriguez-Artalejo F, Bobak M. Mediterranean diet and physical functioning trajectories in Eastern Europe: Findings from the HAPIEE study. PLoS One. 2018;13(7). |  | X |
| Struijk EA, Guallar-Castillón P, Rodríguez-Artalejo F, López-García E. Mediterranean Dietary Patterns and Impaired Physical Function in Older Adults. J Gerontol A Biol Sci Med Sci. 2018;73(3):333-9. | X | X |
| Talegawkar SA, Bandinelli S, Bandeen-Roche K, Chen P, Milaneschi Y, Tanaka T, Semba RD, Guralnik JM, Ferrucci L. A higher adherence to a mediterranean-style diet is inversely associated with the development of frailty in community-dwelling elderly men and women. J Nutr. 2012;142(12):2161-6. | X | X |
| Van Zant RS, Conway JM, Seale JL. A moderate carbohydrate and fat diet does not impair strength performance in moderately trained males. J Sports Med Phys Fitness. 2002;42(1):31‐7. | X | X |
| Yokoyama Y, Nishi M, Murayama H, Amano H, Taniguchi Y, Nofuji Y, Narita M, Matsuo E, Seino S, Kawano Y, et al. Dietary variety and decline in lean mass and physical performance in community-dwelling older Japanese: A 4-year follow-up study. J Nutr Health Aging. 2017;21(1):11-6. |  | X |
| Zhu J, Xiang YB, Cai H, Li H, Gao YT, Zheng W, Shu XO. A Prospective Investigation of Dietary Intake and Functional Impairments Among the Elderly. Am J Epidemiol. 2018;187(11):2372-86. |  | X |

^1^ Van Elswyk ME, Teo L, Lau CS, Shanahan CJ. Dietary Patterns and the Risk of Sarcopenia: A Systematic Review and Meta-Analysis. Curr Dev Nutr 2022;6(5):nzac001.
